# Cathode active materials using rare metals recovered from waste lithium-ion batteries: A review

**DOI:** 10.1016/j.heliyon.2024.e28145

**Published:** 2024-03-20

**Authors:** Yusuke Abe, Ryoei Watanabe, Tatsuya Yodose, Seiji Kumagai

**Affiliations:** a Joint Research Center for Electric Architecture, Akita University, Tegatagakuen-machi 1-1, Akita, 010-8502, Japan; b Environmental Protection Laboratory, DOWA ECO-SYSTEM Co., Ltd., 65-1 Omoriyama-shita, Hanaoka, Odate, 017-0005, Japan; c Department of Mathematical Science and Electrical-Electronic-Computer Engineering, Akita University, Tegatagakuen-machi 1-1, Akita, 010-8502, Japan

**Keywords:** Lithium-ion battery, Cathode, Active material, Rare metal, Recycle, Battery waste

## Abstract

Large-scale lithium-ion batteries (LIBs) are overtaking as power sources for electric vehicles and grid-scale energy-storage systems for renewable sources. Accordingly, large amounts of LIBs are expected to be discarded in the near future. Recycling technologies for waste LIBs, particularly for valuable rare metals (Li, Co, and Ni) used in cathode active materials, need to be developed to construct continuous LIB supply chains. Various recovery methodologies, such as pyrometallurgy, hydrometallurgy, and direct recycling, as well as their advantages, disadvantages, and technical features, are briefly introduced. We review the electrochemical performances of these cathode active materials based on recycled rare metals from LIB waste. Moreover, the physicochemical properties and electrochemical performance of the cathode active materials with impurities incorporated during recycling, which have high academic significance, are outlined. In hydrometallurgy-based LIB recycling, the complete removal of impurities in cathode active materials is not realistic for the mass and sustainable production of LIBs; thus, optimal control of the impurity levels is of significance. Meanwhile, the studies on the direct recycling of LIB showed the necessity of almost complete impurity removal and restoration of physicochemical properties in cathode active materials. This review provides a survey of the technological outlook of reusing cathode active materials from waste LIBs.

## Highlights

• Recycling methods for rare metals in waste Li-ion batteries were briefly explained.
• Advantages, disadvantages, and outlooks for each recycling technology were outlined.
• Influence of impurities in cathode active materials was described.
• Future perspectives of impurities for the recovered cathodes were outlined.

## Graphical abstract

**Figure:**
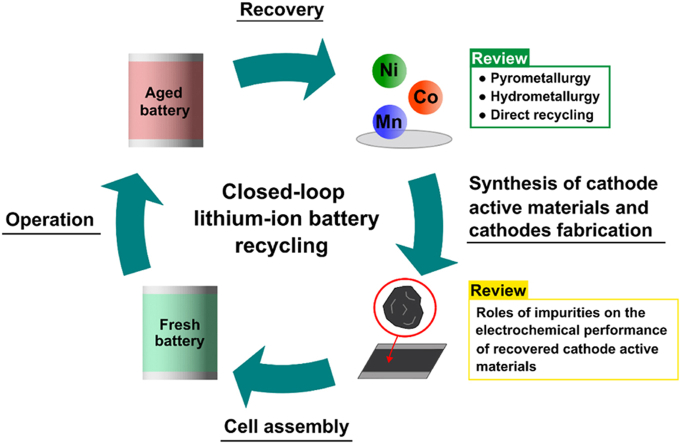

## 1 Introduction

Resource depletion, air pollution, and greenhouse warming are crucial environmental challenges; consequently, the automotive industry is currently addressing these issues by developing electric and plug-in hybrid vehicles that use clean energy and have lower exhaust emissions [1,2]. Electrically powered vehicles require high-capacity, high-power, energy-storage devices, such as lithium-ion batteries (LIBs) [3,4]. LIBs are also installed as stationary energy storage units in grid systems that augment renewable energy sources [5]. The expansion of large-scale LIB applications will inevitably lead to increased demand that requires a stable supply of construction materials and the mass production of LIBs.

**Fig. 1:**
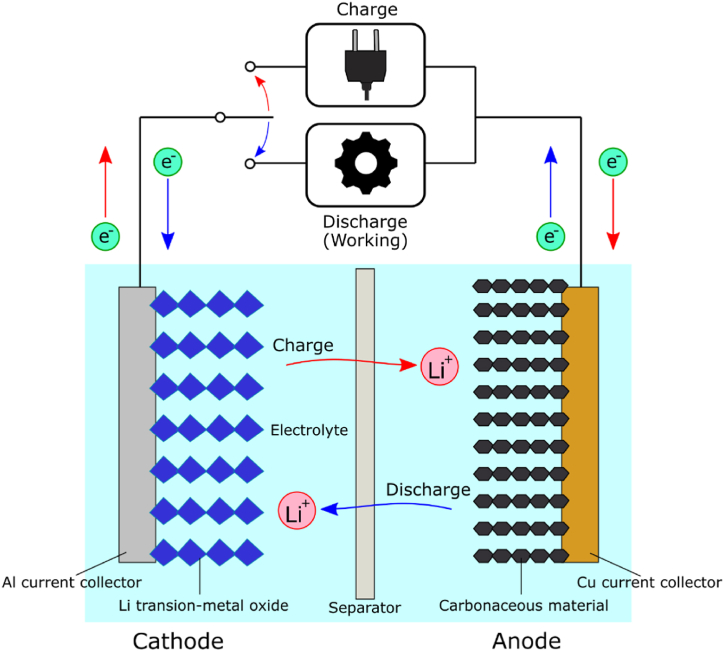
Component materials and charging–discharging mechanism in an LIB cell.

The component materials and charging–discharging mechanism of an LIB cell are shown in Fig. 1. One or more active materials are typically used in the cathode and anode, where oxidation and reduction reactions, respectively, occur during charging and discharging. An LIB enables repeatable charging–discharging at a high operating cell voltage by exchanging Li ions between the cathode and anode, thereby acting as an energy supplier for various applications. A lithium transition-metal oxide cathode and a carbonaceous anode are generally combined in an LIB cell to achieve stable and superior battery performance (i.e., high cell voltage, high energy density, long cycle life, and low self-discharge) [6,7]. The electrolyte and separator play roles that transport Li ions between the two electrodes and prevent direct physical contact (and electrical short-circuiting) between the electrodes, respectively. The lithium transition-metal oxide in a cathode contains certain amounts of rare metals (for example, Li, Co, and Ni) [8] that are associated with some problems, including limited production, poor supply, and high sourcing costs. Therefore, purveying these rare metals required for LIB cathodes is becoming difficult.

**Fig. 2:**
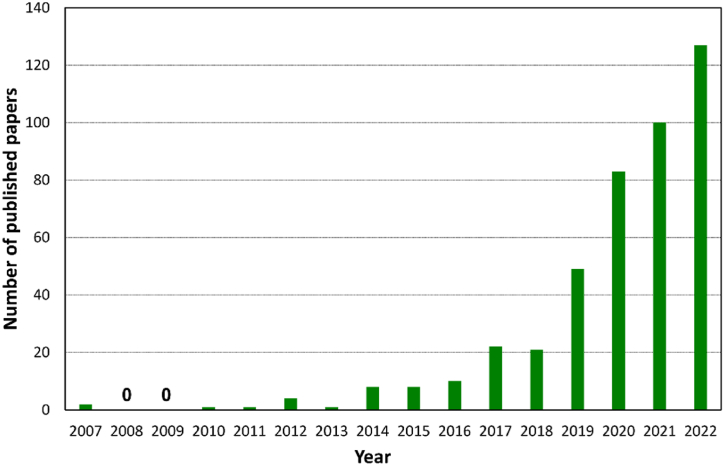
Number of published papers containing “LIB”, “cathode”, and “recycling” keywords according to Scopus.

Recycling rare metals previously used in waste LIBs is attracting increasing attention in terms of the effective reutilization of valuable resources. Common recycling methods typically involve mechanical, thermal, and chemical processing [9,10]. However, these recycling processes have disadvantages, including electrical shock hazards during predischarging, accidental explosions during disassembly, and the use of manual operations [11,12]. A large quantity of waste LIBs (end-of-life and defective products) are forecast to be discharged in the coming decades, irrespective of application and size. Technological innovations focusing on automating recycling processes with high safety and recovery efficiencies along with being economical and eco-friendly are required to meet the future disposal requirements of large quantities of waste LIBs. Interestingly, studies associated with recycling waste LIBs have dramatically increased in recent years, according to Scopus (Fig. 2). Researchers are becoming interested in developing functional and novel recycling processes and characterizing cathode active materials resynthesized from the recovered rare metals.

This review presents a summary of waste-LIB recycling technologies and LIB cathodes using recycled rare metals from waste LIBs. During waste-LIB recycling based on pyrometallurgy and hydrometallurgy, metallic and nonmetallic impurities derived from LIB packs, its outer covering, and used chemical-reagent residues are inevitably incorporated. Direct recycling enables simple material preparation on cathode active materials; however, carbon black, binder, graphite, electrolyte, and charging–discharging byproducts are contaminants. The role of these impurities on the electrochemical performances of the LIB cathodes was intensively discussed. We aimed to review the advantages and disadvantages of reusing rare metals remaining in waste LIBs and the electrochemical performance of corresponding prepared materials for use in LIB cathodes. This paper is outlined as follows. First, current cathode active materials and their electrochemical properties are briefly presented, along with the recycling processes for recovering the rare metals remaining in waste LIBs. Second, the challenges associated with material preparation using hydrometallurgy- and direct recycling-based waste-LIB recycling methods, and the electrochemical performance of active materials prepared for use in LIB-cathode applications are outlined. Finally, future perspectives on the recycling of the cathode active materials of LIBs are described.

## 2 Existing active materials for LIB cathodes

An LIB cell has a specified Li-storage capacity that is limited by the chemistry of its cathode active material because the Li in such a material is a finite Li source within the LIB [13]. The working potential, current-density dependence (rate performance), and cyclability also depend on the selected cathode active materials. Therefore, the choice of cathode material is important to achieve superior charging and discharging performance in an LIB cell [14,15]. Lithium transition-metal oxides are commonly employed as cathode active materials and are chosen from various inorganic oxidized materials, with layered-type (LiCoO_2_, LiNiO_2_, and LiNi_1−x−y_Co_x_Mn_y_O_2_ (NCM) [16,17]), spinel-type (LiMn_2_O_4_ and LiNi_0.5_Mn_1.5_O_4_ [[18], [19], [20]]), and olivine-type (LiFePO_4_ [21,22]) crystalline materials typically selected.

Among layered materials, ternary nickel-cobalt-manganese-oxide-based (NCM-based) cathodes are favorably used in LIBs for vehicle applications [23,24]. The electrochemical performance of NCM depends on its chemical composition. In particular, the Li, Ni, Co, and Mn ratios affect the cathodic performance of the active material. The Li in a layered material (LiCoO_2_ and LiNiO_2_) determines the Li-storage capacity per unit mass (specific capacity), internal resistance, and electrochemical behavior during lithiation and delithiation [25]. Meanwhile, Ni elevates the specific capacity, Co improves electronic conductivity, and Mn helps maintain a stable crystalline structure while strengthening cyclability [14,17,26]. The LiNi_1/3_Co_1/3_Mn_1/3_O_2_ (NCM111) cathode formed using an equal amount of each of the three transition metals (Ni, Co, and Mn; *x* = *y* = ∼1/3) operates with good cyclability, is highly thermally stable, and exhibits a specific capacity of 150–160 mAh g^−1^, with a charge cutoff voltage of 4.3 V vs. Li/Li^+^ [27]. High-Ni-containing NCMs (LiNi_0.5_Co_0.2_Mn_0.3_O_2_ [NCM523] and LiNi_0.6_Co_0.2_Mn_0.2_O_2_ [NCM622]) formed by increasing the Ni content to 0.5 and 0.6, respectively, showed higher specific capacities than NCM111 [28,29].

Although the approach of increasing the Ni content leads to an NCM with high capacity, which highlights its electrode-level specific energy, further enhancement of the Ni content (>0.8) led to poor cyclability, low thermal stability, and Li/Ni cation mixing in the active material [30]. Several researchers have attempted to overcome these problems through judicious material design (such as surface modification, cation doping, and unique particle modification) and the use of functional materials (electrolyte additives and binders) [[31], [32], [33]]; such strategies have produced NCM811, which exhibits superior cathodic performance. However, commercializing LIBs that use the NCM811 cathode is expected to take time; consequently, NCM cathodes with low-to-average Ni contents are broadly acceptable for use in existing LIBs owing to their satisfactory cathodic performance and cost-effectiveness compared to LiCoO_2_ cathodes.

Reducing material costs, along with improving LIB-cathode performance, is another important issue. Early commercialized LIBs employed LiCoO_2_ cathodes [34]; however, Co is expensive and scarce but is abundantly contained in LiCoO_2_ as battery grade products. Cathode materials composed of rare metals cost twice as much as carbon- or Si-based anode materials, with the cathode accounting for a high percentage of the entire cost of an LIB cell [35]. Meanwhile, rare metals are seriously restricted in terms of material supply owing to limited production in various countries and associated low yields. Considering the high costs and supply issues, research on the development of next-generation cathode active materials has focused on low-Co-content or Co-free materials that are more cost-effective. NCM is a realistic choice for reducing material costs because it can be formulated with lower amounts of Co through replacement with other transition metals, such as Ni and Mn. LiFePO_4_, which has a slightly lower working potential and energy density than a layered material, is also an attractive option for low-cost LIBs [36,37]. However, LiFePO_4_ requires preprocessing by carbon coating, producing smaller particles, and effective cation doping owing to its low electronic conductivity derived from its crystalline structure (olivine structure) and its Fe content [38]. Preprocessed LiFePO_4_ exhibits a superior current-rate dependence (time response), admissible specific capacity, and excellent cyclability as an LIB cathode material. Attempts to develop high-performance and low-cost cathode active materials have subsequently been explored on an ongoing basis.

Recent progress in cathode-chemistry has predominantly focused on enhancing the specific capacities and energies of LIBs. Elevating the working cathodic potential (>4 V vs. Li/Li^+^) is also significant for cathode active materials in high-energy-density LIBs. High Ni- or Li-content materials with layered structures have attracted considerable attention for their high capacity in LIB cathodes. Cathodes using Ni- and Li-rich materials facilitate Li-ion intercalation/deintercalation with a specific capacity 1.25−1.50 times higher than that of low-Ni-content materials such as NCM111 [39]. The representative notation of Li-rich layered material is *x*Li_2_MnO_3_·(1−*x*)LiMO_2_ (M = Mn, Ni, Co, Cr, and others). When the working potential increases above 4.7 V, a significantly high specific capacity (>250 mAh g^−1^) is obtained for Li-rich cathodes [35,40]. However, voltage fading and a decrease in Li-ion mobility are observed during the operation of the LIB cathodes. This resulted from the rearrangement of bulk and surface structures in the materials, leading to unfavorable current rate performance and severe capacity fade during cycling. Effective solutions (e.g., cation doping, surface coating, nanosized particles) are proposed to overcome the above-mentioned challenges [35,41]. Binary Ni-containing materials based on layered structures are promising for high-capacity LIB cathodes. Ni and other transition metals mainly constitute binary materials, denoted as LiNi_1−*x*_M_*x*_O_2_ (M = Co, Mn, and others). LiNi_0.5_Mn_0.5_O_2_ has a theoretical capacity of 280 mAh g^−1^ as LIB cathode active materials, and the lithiation and delithiation capacity are dominantly obtained via the Ni^2+^/Ni^4+^ redox reaction. In LiNi_0.5_Mn_0.5_O_2_, the presence of Mn^4+^ contributes to the stabilization of the crystalline structure, while Mn^4+^ is electrochemically inactive during oxidation-reduction under cathodic operation. LiNi_*x*_Co_1−*x*_O_2_ has been proposed for both performance improvement for specific capacity and structure stabilization of the LIB cathodes. The higher the Co content in LiNi_*x*_Co_1−*x*_O_2_, the more stable the crystalline structure. Substituting Ni with Co at *x* = 0.8 in such a chemical formula, a high capacity and favorable reversibility were obtained in the lithiation/delithiation process in a previous study [42]. Other studies reported the improvement of cathodic performances of LiNi_*x*_Co_1−*x*_O_2_ by the addition of small amounts of Al and Mg [41,43,44]. High-voltage materials such as LiNi_0.5_Mn_1.5_O_4_ have driven researchers to develop high-energy and high-power-density LIBs. LiNi_0.5_Mn_1.5_O_4_ with a spinel structure has garnered considerable attention owing to their high working cathodic potential (4.6−4.8 V vs. Li/Li^+^), excellent rate performance, and good cyclability [45]. In LiNi_0.5_Mn_1.5_O_4_, the working potential and lithiation capacity are derived from the Ni^2+^/Ni^3+^/Ni^4+^ redox reaction. The intrinsic crystalline framework determines the dimension of the Li^+^ diffusion channel for cathode active materials. The Li^+^ diffusion channel of the spinel-based materials (e.g., LiMn_2_O_4_ and LiNi_0.5_Mn_1.5_O_4_) is three-dimensional, whereas that of layered-based materials (e.g., LiNiO_2_ and LiCoO_2_) is two-dimensional. The higher the dimension of the Li^+^ diffusion channel, the higher the utilization of each active material used in LIB cathodes [19]. The utilization depends on the dimension of the Li^+^ diffusion channel and Li^+^ diffusion length, which arises from the crystalline structure and Li^+^ diffusion coefficient of the cathode active materials. Under the severe conditions required for considerably short charging and discharging times, the cathode active materials with higher dimensions of the Li^+^ diffusion channel can deliver an effectively high specific capacity, and thus, the current-density response at high current densities (shorter charging/discharging time) is superior. Spinel materials such as LiNi_0.5_Mn_1.5_O_4_ have the potential as next-generation cathode materials for high-power and high-energy density LIBs. The LiNi_0.5_Mn_1.5_O_4_ cathode is frequently combined with Li_4_Ti_5_O_12_ and a hard carbon anode to fabricate high-power-intended LIBs with relatively high energy densities [19,46].

## 3 Recycling waste LIBs

### 3.1 Overview

Dramatically, higher future demands for LIBs are forecast in various sectors (such as electronic devices, transportation, and stationary energy storage) [47,48]. Although the continuous production and use of LIBs contribute to the effective use of renewable energy and widespread uptake of zero-emission vehicles, large amounts of spent LIBs will continue to be discharged over many years in the future. Furthermore, the production of some defective products during LIB production needs to be considered. These batteries still contain valuable metals, such as Li, Ni, and Co; hence, developing recycling and recovery technologies is an important solution for the economical and circular supply of materials, considering the highly competitive nature of the rare metal market [49,50]. Consequently, effectively collecting the metals from waste LIBs and using them in the subsequent production of LIBs are essential. LIB-recycling methods are classified as i) pyrometallurgy and hydrometallurgy and ii) direct recycling according to the method used to process the cathode active material present in the waste.

Regardless of the recycling method, waste LIBs generally require deactivation (pre-discharging), dismounting, and mechanical separation to safely collect their metal components. The mechanical pretreatments enable the separation of unwanted matter in the LIB waste as well as the condensation of target metals recovered by recycling. Deactivation and mechanical disassembly can result in electrical shocks and heating accidents during preprocessing. This carries risks owing to difficulties associated with the handling and disassembly of commercial LIBs, mostly involving manual procedures. Waste LIBs are typically predischarged by immersion in a NaCl solution or high-speed shredding in cryogenic nitrogen, which are well-known methods for liberating the residual capacity of an LIB [51,52]. In particular, high-speed shredding is an effective approach that avoids the disassembly process and ensures safety owing to the inert N_2_ atmosphere and automation. The dismounting process generally requires intensive manual operations, whereas the separation process can be semi- or fully automated. In the future, high recovery efficiency, minimal effort, and high safety, all in one automated process, will require the recycling process to be performed in an industrial arena.

### 3.2 Pyrometallurgy and hydrometallurgy

The recycling process enables the collection of various metals from damaged cathode materials in waste LIBs and their reproduction as fresh materials. Pyrometallurgy involves roasting, calcination, and pyrolysis at high temperatures [53], with intermediate products (alloys, slags, and gases) generated pyrometallurgically during waste-LIB recycling [9,[54], [55], [56]]. Alloys typically contain Co, Cu, Ni, and Fe, whereas slags contain Li, Al, Mn, Si, and Ca [54,57]. Pyrometallurgy is a promising method with a large disposal capacity and has been favorably adopted by major recycling companies [58,59].

The heat-treatment temperature is an important factor for removing impurities (carbon black, polyvinylidene difluoride (PVDF) binder, electrolyte, and organic solvents) during pyrometallurgy, as it ensures efficient follow-up processing [53,[60], [61], [62]]. Pyrometallurgical treatment is favorably combined with mechanical treatments and should be performed at an earlier stage in waste-LIB recycling. High concentrations of target metals are achieved by pyrometallurgy at suitable temperatures and by mechanical sieving using processes such as magnetic selection and gravimetric concentration. The separated materials, including the concentrated metals, are then processed in a subsequent step (hydrometallurgical treatment).

Hydrometallurgy is an effective method owing to its low hazardous gas emissions, secure recovery of specified metals, and high recovery efficiencies [[63], [64], [65]]. Metals concentrated by preprocessing undergo hydrometallurgical methods to selectively recover metals needed for the synthesis of lithium transition-metal oxides (cathode active materials). The starting materials for hydrometallurgy are in a complex form; most of them are mixed matter coated on an Al foil as a cathode current collector. The mixtures are generally obtained by disassembling waste LIB, separating the cathode component, and peeling from the current collector. The separation methods require considerable effort for manual fusion and certain chemical reagents in the peeling process. To minimize manual effort and risk, pyrolysis residue, known as black mass, treated simply by pyrometallurgy is set to starting material for the subsequent hydrometallurgical process [66]. Regardless of the type of starting materials, target metals are included in the mixture (e.g., coating layer peeled from Al foil, and black mass), as they are necessary to ensure separation and selective recovery. Mixtures including the metals undergo lixiviation (leaching) using inorganic or organic acids. Leaching metals into a solvent transforms the metals into metallic ions. The dissolved metallic ions are then collected using solvent extraction, chemical precipitation, and electrolytic deposition, leading to the solidification of metal compounds suitable for subsequent processing.

Automotive LIBs for electrical vehicles and plug-in hybrid vehicles generally recruit ternary-mixed cathodes—NCM and lithium nickel cobalt aluminum oxide (NCA)— which are composed of multi-metals such as Li, Ni, Co, Mn, or Al [67]. The battery systems comprise a variety of metallic elements (e.g., Li, Ni, Co, Mn, Al, Cu, Fe, Ca, and Mg) as the cathode active material, electrode current collectors, conductive bus bar, battery protective casing, and others. For this reason, the recovery of the desirable metals from such complex mixtures is labor-intensive but essential for circulative material preparation. Significant works focusing on the recovery and purification of single or multiple metals from the materials obtained from waste-LIBs (or parts of them) have been conducted in recent years [66,68,69]. During the resynthesis process, F and P, nonmetallic impurities derived from Li salt in organic electrolytes, are likely to remain in the preparing material. The inclusion of other nonmetallic elements (e.g., Na, Cl, and S) from the chemical reagents used for hydrometallurgical treatments is also a concerning issue. The removal of the impurities can be achieved by selective precipitation by controlling various factors such as the pH in leachate, solvent extraction, ion-exchange removal, and washing with distilled or ion-exchanged water. However, under commercial mass processing and reasonable cost-effectiveness, perfect removal of the contaminants is still challenging. Thus, researchers have recently extensively evaluated the electrochemical performance of active cathode materials containing impurities [57,[70], [71], [72]].

### 3.3 Direct recycling

Direct recycling is another method that facilitates the utilization of valuable metals in waste LIBs [[73], [74], [75], [76]]. It involves liberation and reactivation processes that rely on chemical, mechanical, or thermal treatment. First, the target cathode active material is typically coated on an Al foil current collector. The compound must be separated from the Al foil and then freed from other electrode components, such as the conductive agent and polymer binder, during the liberation process. The compound is typically peeled from the Al foil by sintering under inert conditions or immersion in an organic solvent (such as *N*-methylpyrrolidone, *N,N*-dimethylformamide, and *N,N*-dimethylethanamide) [75,76], after which the conductive agent, binder, and electrolyte residues are removed by additional thermal treatment. Reactivation is achieved using any Li source or Li-containing residue in the separated materials [73,77]; here, adding a Li source and implementing solid-phase chemistry actively supplement the Li in damaged materials recovered from cathode components.

Direct recycling is advantageous compared to the hydrometallurgical method owing to the ready availability of recycled materials, fewer processing steps, and lower environmental burden, making it an economical and environmentally friendly method [78]. However, any residues, such as charging–discharging byproducts and unreacted materials from the reactivation process, will likely result in contamination during direct recycling. Consequently, how these residues affect the electrochemical performance of the obtained products needs to be extensively investigated.

## 4 Electrochemical performance of recycled cathode active materials

Table 1 lists the material specifications and electrochemical performance of cathode active materials resynthesized from recycled rare metals. The physical and electrochemical properties of the cathode active materials with rare metals recycled from waste LIBs have been concurrently characterized following metal recovery and material preparation during waste-LIB recycling. In this Section, we collected research papers published within the last 11 years.

**Table 1:** Material specifications and electrochemical performance of cathode active materials resynthesized from recycled rare metals.

Material specification		Starting materials	Pretreatment and metal-recovery methods	Performance			Ref.
Target chemical composition	Mainly included impurities			CAP_MAX_^a^ [mAh g^−1^]	Current rate	Cycling	
LiNi_1/3_Co_1/3_Mn_1/3_O_2_	2.54 at% Al, 0.16 at% Cu, 4.21 at% F, 2.11 at% S	Spent LIBs used for laptop and EV applications	Mechanical pretreatment Acid leaching Precipitation	∼136 @ 0.1C	Improved	Deteriorated	[79]
LiNi_1/3_Co_1/3_Mn_1/3_O_2_	0.57 mass% Al	Spent NCM-based LIBs	Mechanical pretreatment Acid leaching Precipitation	∼115 @ 1C	Sustained	Deteriorated	[80]
LiNi_1/3_Co_1/3_Mn_1/3_O_2_	4 mol% Al	Spent LCO-based LIBs	Mechanical pretreatment Acid leaching Sol-gel method	154 @ 0.2C	Improved	Sustained	[81]
LiCoO_2_	Specified small amounts of Cu	Spent LIBs used for portable applications	Mechanical pretreatment Acid leading Precipitation	128 @ 0.03C	Improved	Improved	[82]
LiNi_1/3_Co_1/3_Mn_1/3_O_2_	0.05 mol% Mg	Spent LIBs	Thermal and mechanical pretreatment Acid leaching Precipitation	∼155 @ 0.5C	–	Deteriorated	[83]
LiNi_1/3_Co_1/3_Mn_1/3_O_2_	Almost completely removed	Spent LIBs	Mechanical pretreatment Acid leaching Precipitation	–	Improved	Improved	[84]
LiNi_1/3_Co_1/3_Mn_1/3_O_2_	1.3 mol% Al, 1.7 mol% Cu, 1.0 mol% Fe	Spent LIBs used for PHEV applications	Thermal and mechanical pretreatment Acid leaching Precipitation	121 @ 0.1C	Sustained	Deteriorated	[66]
LiNi_1/3_Co_1/3_Mn_1/3_O_2_	Almost removed	End-of-life LIBs	Mechanical pretreatment Acid leaching, and Ni, Mn, and Co composition controlling Precipitation	158 @ 0.1C	Improved	–	[69]
LiNi_1/3_Co_1/3_Mn_1/3_O_2_	0.01 mass% Fe, 0.03 mass% Ca, 0.01 mass% Cu, 0.02 mass% Zn, 0.01 mass% Mg, 0.03 mass% Na	Spent LIBs including LiCoO_2_, LiNiO_2_, LiMn_2_O_4_, NCM, and LiFePO_4_ cathodes	Mechanical and chemical pretreatment Acid leaching, and Ni, Mn, and Co composition controlling	149 @ 0.2C	Sustained	Sustained	[85]

a Maximum specific capacity during delithiation in the initial cycle.

Secure recovery and good rare-metal recovery rates are required to recycle waste LIBs effectively. If the requirements are met in the LIB recycle industry at home, a domestically stable supply chain for rare metals could be structured along with effective utilization of them without mining in the earth. Besides the advantages of waste-LIB recycling, attention should be paid to impurity removal for material production with high-quality and comparable or superior electrochemical performances as LIB cathodes. Ma et al. and Beak et al. reported the near-complete removal of recycling contaminants, leading to specific capacities that are nearly identical to those of the initial materials [79,84]. However, how specific amounts of metallic or nonmetallic impurities in a recycled cathode active material affect the LIB cathode remains unknown.

Metallic and nonmetallic impurities (such as Al, Cu, Fe, Mg, Na, F, S, and Cl) contaminate reproduced cathode active materials during recycling. A small amount of an Al dopant improved the current rate capability and stabilized the crystal structure while decreasing the maximum specific capacity [[86], [87]]. By contrast, excessive amounts of Al in an NCM material led to cycling performance degradation, as confirmed through doping studies [79]. The incorporated Al is also a structural modifier that affects secondary particle formation during synthesis, irrespective of the level of doping [80,81]. Furthermore, a lower cation-mixing frequency was observed in the crystal structure of the cathode active material. LiCoO_2_-based cathodes with Cu impurities exhibited superior rate and cycling performance, albeit with slightly lower acquired specific capacities [82]. Cu contamination is disadvantageous during material synthesis and cathodic operation because Co coprecipitates during impurity removal, forming agglomerated particles, and Cu dissolves and is deposited along with Mn at the anode surface during cell operation [[82], [88]]. Small quantities of a Fe dopant effectively enhanced the rate and cycling performance of an NCM-based cathode owing to the improved crystal structure obtained when Fe occupies Li sites [[72], [89]]. However, impurity-containing NCM-based cathodes showed inferior electrochemical performance when excessive amounts of Fe, Cu, and Al were included [87]; these impurities were derived from the cathode and anode current collectors, body frames, bus bars, and other sources.

Other impurities derived from metal-recovery processes also influence the material preparation and electrochemical performance of recycled cathode active materials [[79], [90], [91], [92], [93]]. For example, Na is a frequent contaminant generated via cell deactivation using NaCl solution and hydroxide precipitation for cathode precursor production using NaOH solution. Na affects the electrochemical performance of an LIB cathode without inhibiting crystal-structure growth. Small amounts of Na in resynthesized cathode active materials confer good rate and cycling performance and less cation mixing, adversely affecting cathodic performance [[90], [91]]. Meanwhile, the inclusion of nonmetallic F and P improved the cathodic performance of synthesized products [78]; a small amount of F in the cathode active material led to good performance, which may explain the effectiveness of a few F dopants. However, excessive F affects the crystalline morphology (e.g., hole generation in the structure) and deteriorates performance [92]. More F is present on the surfaces of synthesized particles. A large amount of F promotes the formation of an SEI film between the cathode and electrolyte, preventing transition-metal ions (Mn and Cu) dissolved in the electrolyte from depositing on the anode surface during full-cell operation [[78], [88]]. Hence, eliminating the remaining electrolytes containing Li salts and the degradation byproducts formed in charging–discharging cycles is essential during recycling.

Sulfuric acid (H_2_SO_4_), which is commonly used in the industry, dissolves valuable metals during the hydrometallurgical leaching process; consequently, S residues are present in the final products [51]. Beak et al. studied the effect of nonmetallic impurities (F and S) on the electrochemical performance of NCM111 cathodes [79]. The incorporation of ∼2 at% S and ∼4 at% F negatively affected performance (lithiation/delithiation specific capacity and cyclability during Li-ion insertion and extraction). Therefore, extensive elimination of recycling residues, such as Na, Cl, and S, is important during recycling. However, extreme impurity removal frequently lowers the recovery efficiency of target metals, and thus, the minor metals are recovered in the recycling process. Some studies have reported that the addition of hydrates and metallic salts including desirable metals was attempted to compensate for the metal loss caused by impurity removal [[69], [85]]. Although the prepared cathode active materials exhibited comparable or superior electrochemical performances, the recycling and material preparation processes require additional chemical processes using certain chemical reagents. This results in the consumption of new reagent products containing mined rare metals, and increases the total cost and environmental burden of the waste-LIB recycling system.

The material specifications and electrochemical performances of cathodic active materials recovered via direct recycling are briefly summarized in Table 2. Direct recycling simply recovers degraded cathode active materials from waste LIBs. In the cathode active materials, the electrode constituent materials (carbon black, binder, current collectors of Al and Cu), graphite, electrolyte, and charging–discharging byproducts are mixed as unfavorable contaminants. The binders, electrolytes, and residues can be removed by dissolving in an organic solvent such as dimethyl carbonate, *N*-methylpyrrolidone, and dimethyl acetamide [76,77]. Higher temperatures (>410 °C) ensure that the decomposition of PVDF-based binders has little effect on the crystalline structure and maximum specific capacity of the LiCoO_2_-based cathode [94]. Pyrolytic residues based on PVDF and CMC/SBR binders provided the cathode active materials with high electron conductivity or reduction ability [[75], [94]]. Meanwhile, the remaining binder requires to be removed, as the produced pyrolytic residues are likely to act as conductive agents. It is recommended that carbonaceous residues, such as carbon black and graphite, also be removed to sustain and improve the electrochemical performance of the LIB cathodes. The removal of carbonaceous materials is readily achieved by high-temperature heating under O_2_ or air. Their presence causes the deterioration of electrochemical performances (increasing internal voltage drop, lowering specific capacity, deteriorating ionic conductivity) of the recycled cathode active materials [[77], [94], [95]]. When the cathode active materials recycled by direct recycling are used in the next LIB cathodes, along with the reactivation process, the removal of impurities in the form of LIB-constituent-material residues is also necessary for the restoration of degraded cathode active materials.

**Table 2:** Material specifications and electrochemical performance of cathode active materials recovered by direct recycling.

Material specification		Starting materials	Pretreatment and metal-recovery methods	Performance			Ref.
Target chemical composition	Mainly included impurities			CAP_MAX_^a^ [mAh g^−1^]	Current rate	Cycling	
LiFePO_4_	Not analyzed (Possible contaminants: electrode-derived CMC/SBR and carbon black residues)	Scraps discarded by LFP cathode production	Thermal and mechanical pretreatment	135 @ 0.1C	Sustained	–	[75]
LiFePO_4_	Not analyzed (Possible contaminants: LFP-derived decomposition products)	Spent LFP-based LIBs used for EV applications	Mechanical and chemical treatment Thermal treatment	∼130 @ 0.2C	Improved	Sustained	[74]
LiNi_1/3_Co_1/3_Mn_1/3_O_2_	0.003 at% Al (Possible contaminants: electrode-derived PVDF and carbon black residues)	Spent power-type LIBs with 2000 cycles	Mechanical and thermal pretreatment Mechanochemical treatment with Li additives Thermal treatment	165 @ 0.2C	Improved	Sustained	[73]
LiNi_1/3_Co_1/3_Mn_1/3_O_2_	Not analyzed for metallic impurities (Possible contaminants: Al, Li-containing compounds [LiF and Li_2_CO_3_])	Spent NCM-based LIBs	Mechanical pretreatment Thermal treatment with re-lithiation by surface Li residue	170 @ 0.1C	Improved	Improved	[77]
LiNi_1/3_Co_1/3_Mn_1/3_O_2_	Almost removed	End-of-life prismatic LIBs with 20 Ah	Mechanical pretreatment Hydrothermal treatment for re-lithiation Washing with distilled water Thermal treatment	155 @ 0.1C	Sustained	Sustained	[94]
LiCoO_2_	Almost removed	Spent LIBs for a laptop	Mechanical pretreatment Molten-salt, chemical and thermal treatments	149 @ 0.2C	Sustained	Sustained	[95]

a Maximum specific capacity during delithiation in the initial cycle.

Reactivation plays an important role in repairing the crystalline structure and compensating for active Li in the degraded cathode active materials. Various methods of reactivation have been proposed; for example, mechanochemical, thermal, hydrothermal, and molten-salt treatments [[73], [77], [94], [95], [96]]. Regardless of the types of the reactivation method, the Li source (e.g., Li_2_CO_3_, LiOH) is required to be added to the material, and then treated with a certain method depending on the Li source. Another approach has been attempted by using Li residue (e.g., Li_2_CO_3_ and LiF) as the Li source on the surface of the cathode active materials [77]. As the Li is recovered by reactivation, cathodes using the recycled materials exhibited comparable or superior specific capacity than that using pristine materials.

In hydrometallurgy-based waste-LIB recycling, some impurities contaminate at the atomic level in the resynthesized cathode active materials. The cathode active materials comprise indispensable components (lithium transition-metal oxides) and certain amounts of metallic and nonmetallic contaminants. The synthesized products contaminated with excess amounts of impurities exhibit unstable and faulty crystal structures and inferior performances. By contrast, trace amounts of single/multiple impurities not only sustain electrochemical performance but also positively impact the performance. Previous studies have demonstrated that lower impurities lead to higher lithiation and delithiation-specific capacities [79,84]. Furthermore, small amounts of impurities can lead to robust crystal structures that deliver superior electrochemical performance and stable LIB-cathode operation. The complete removal of impurities is not realistic for constructing the hydrometallurgy-based waste-LIB recycling system, although it can be achieved through extreme processing when effort, cost, and environmental burden are not considered.

In direct recycling, the recovered cathode active materials generally contained impurities in the form of residues (carbon black, binder, graphite, electrolyte, and charging–discharging byproducts). These impurities existed at the surface of the cathode active materials and in the mixtures containing the main salvaged materials of the degraded cathode active materials. They can be removed by mechanical, chemical, and thermal treatments according to their chemical properties. Following impurity removal, the degraded cathode active materials are reactivated with a Li source and exhibit sustained or superior properties (crystalline structure, chemical composition, and LIB cathodic operation) compared with that of pristine materials [[94], [95]]. Compared with hydrometallurgy-based waste-LIB recycling, direct recycling essentially has the advantages of reducing energy consumption, avoiding toxic chemical treatments, and demonstrating rapid material preparation. Hence, LIB recycling with direct recycling is recommended to produce high-quality cathode active materials with extremely low amounts of impurities, even if it requires one or more additional treatments.

## 5 Summary and future perspectives

As LIB demand is predicted to increase dramatically in the future, the mass production and acquisition of required electrode materials are clear imperatives. LIB recycling technologies are essential for the effective utilization of rare metals, the establishment of a sustainable supply chain for rare metals, and producing newly massive LIBs. This paper reviews representative LIB recycling methods, along with their characteristics and benefits, and the electrochemical performance of impurity-containing cathode active materials prepared using hydrometallurgy or direct-recycling methods.

Existing LIB cathode active materials contain expensive rare metals (Li, Co, and Ni). Considering that the acquisition of such precious resources is highly competitive, continued consumption of large amounts of raw materials through mining is not sustainable from the perspectives of finite natural resources, economic considerations, and geo-environmental impact. Therefore, two major approaches (hydrometallurgy-based and direct-recycling-based waste-LIB recycling) are promising for securely supplying raw materials without additional harvesting. Such approaches contribute to solving serious problems associated with material supply and the costs of LIB cathodes. Hence, recycling technologies that use pyrometallurgy, hydrometallurgy, and direct recycling, and their combination, have effectively utilized rare metals in waste LIBs. Future recycling processes will require highly safe and simple preprocessing methods that ensure high recovery rates for the desired metals and do not rely on manual labor.

As for hydrometallurgy-based waste-LIB recycling, the complete removal of impurities in the recycled products, which requires extreme processing involving many chemical reagents, high costs, and a considerable environmental burden, is not realistic. Trace amounts of single/multiple impurities not only maintain electrochemical performance but also positively improve performance. Thus, in hydrometallurgy-based waste-LIB recycling, we recommend that the inclusion levels of impurities be optimally controlled for use in the next LIB cathode active materials. Direct-recycling-based waste-LIB recycling enables rapid material preparation on cathode active materials; however, other LIB constituent materials and charging–discharging byproducts are contaminants and act as impurities. Considering fewer processing steps and rapid material preparation with the advantages of direct recycling, the impurities should be removed as much possible as, producing high-quality cathode active materials with repaired crystalline structure and chemical composition by reactivation.

## CRediT authorship contribution statement

**Yusuke Abe:** Writing – original draft, Methodology, Investigation, Conceptualization. **Ryoei Watanabe:** Writing – review & editing, Supervision, Methodology, Investigation. **Tatsuya Yodose:** Writing – review & editing, Validation, Methodology, Investigation. **Seiji Kumagai:** Writing – review & editing, Writing – original draft, Validation, Supervision, Project administration, Methodology, Investigation, Funding acquisition, Conceptualization.

## Declaration of competing interest

The authors declare that they have no known competing financial interests or personal relationships that could have appeared to influence the work reported in this paper.
